# Experimental Investigation of the Effect of Nano Silica Fume on Durability of Concrete with Close-Packing Aggregate

**DOI:** 10.3390/ma18174061

**Published:** 2025-08-29

**Authors:** Zilong Ye, Xin Qu, Jiajun Li, Tianhao Ye, Gengying Li, Haiyang Wang

**Affiliations:** 1 Dongguan Development Holdings Co., Ltd., Dongguan 523000, China; yezilong2025@163.com; 2 Guangdong Provincial Transportation Planning and Design Institute Group Co., Ltd., Guangzhou 510623, China; ixinqy@163.com; 3 College of Water Conservancy and Civil Engineering, South China Agricultural University, Guangzhou 510642, China; lijj363@mail2.sysu.edu.cn (J.L.); 15986356141@163.com (T.Y.)

**Keywords:** concrete, close-packing aggregate, nano silica fume, long-term mechanical properties, durability

## Abstract

Achieving the close packing and interlocking of coarse aggregates in concrete enhances the elastic modulus, thereby reducing deformation, and can improve the overall stiffness of concrete structures. This study focuses on reinforcing and toughening concrete with close-packing aggregate with silica fume and micro-steel fibers, and investigates its durability properties, including long-term mechanical performance, water absorption, and sulfate erosion resistance under dry–wet cyclic exposure. The experimental results indicate that the 360-day long-term compressive strength of the concrete reaches up to 109.3 MPa, and the 360-day flexural strength reaches 11.62 MPa. The addition of silica fume effectively reduces the water absorption of concrete with close-packing aggregate and improves its sulfate erosion resistance under dry–wet cycles. The lowest 28-day water absorption rate is 2.41%, and after 150 cycles of sulfate erosion, the compressive strength corrosion resistance coefficient of the concrete can be maintained at up to 68.4%, while the sulfate erosion resistance grade reaches up to KS120. The concrete overall exhibits excellent durability properties. Moreover, this is beneficial for enhancing the concrete’s performance under dry–wet cycles and its resistance to the effects of sulfate attack.

## 1. Introduction

Excessive structural deflection and deformation are prevalent issues in structural engineering [1]. Significant instances include excessive lateral displacement in high-rise buildings and mid-span deflection in long-span prestressed continuous rigid-frame bridges [2,3]. When deformations in concrete structures are poorly controlled, this critically undermines the safety and reliability of the structure [4,5].

It is well known that the stiffness of concrete members and structures determines the degree of deformation in concrete buildings during service. The elastic modulus of concrete is a crucial parameter for evaluating the stiffness of concrete members and guiding structural design [6]. It reflects the relationship between stress and strain in the elastic stage of concrete, serving as an important indicator of the material’s resistance to elastic deformation [7]. Increasing the elastic modulus of concrete can effectively improve the structural stiffness and suppress flexural deformation.

Coarse aggregates form the rigid skeleton of concrete, playing a vital role in resisting deformation under load [8]. The type, size, shape characteristics, and relative content of coarse aggregates in concrete significantly influence the elastic modulus [9,10]. Therefore, in concrete with close-packing aggregate, the densely packed aggregates construct a high-stiffness skeleton, enabling a high elastic modulus of the concrete. It should be noted that concrete with close-packing aggregate exhibits significant brittleness due to its high elastic modulus, necessitating further reinforcement and toughening modifications.

The matrix enhancement of concrete is mainly realized by improving the strength and hardness of the cement mortar matrix, as well as improving the microstructure of the matrix, reducing the pore defects, and realizing a dense matrix, etc. The main methods include adjusting the concrete proportioning parameters, mixing high-activity auxiliary cementitious materials, and using high-performance nano enhancement materials. Poudyal et al. [11] investigated the effect of nano-CaCO_3_ on the mechanical properties of concrete and found that nano-CaCO_3_ can make the matrix denser and then enhances the mechanical properties of concrete. Konsta-Gdoutos et al. [12] addressed the issue of insufficient elastic modulus in high-rise building design by modifying concrete with nanomaterials. Using carbon nanotubes (CNTs) combined with carbon nanofibers (CNFs) can achieve a high elastic modulus. The 28-day elastic modulus of the high-elastic-modulus concrete reached 45.8 GPa, 156% of that of the ordinary reference concrete in the test. Tang et al. [13] used silica fume combined with nano-SiO_2_ to improve the mechanical properties of recycled concrete. The results showed that silica fume and nano-SiO_2_ exhibit synergistic pozzolanic effects, which densify and strengthen the concrete matrix, thereby improving its mechanical properties. Gao et al. [14] studied the effect of silica fume on the interfacial transition zone of recycled concrete and found that silica fume can effectively improve the hardness of the interfacial transition zone of concrete. The 28d compressive strength of concrete with 6% silica fume dosing increased by 9.5% compared to that of the baseline concrete. Ahmad et al. [15] carried out a targeted study on the effect of metakaolin on the mechanical properties and microstructure of concrete. They found that metakaolin was able to refine the pore aperture size in the matrix, and to improve the homogeneity of the matrix. When the dosage of metakaolin was increased, the porosity of the cement matrix was reduced and the porous structure was improved, indicating that metakaolin possesses a matrix enhancement effect. Ambedkar et al. [16] investigated the feasibility of utilizing rice husk ash as a partial replacement for cement to enhance the performance of concrete, and the study showed that rice husk ash, as a high-quality auxiliary cementitious material, which exerted its volcanic ash effect in the cement matrix, strengthened the cement matrix, and the cementitious structure of the cement was enhanced by the fineness of the rice husk ash. And with an increase in the fineness of the rice husk ash, its volcanic ash effect began to be significant.

However, current research rarely investigates the durability of toughened concrete with close-packing aggregate. In reality, during the service life of buildings, structural concrete is required to possess not only excellent mechanical properties to withstand various loads but also good durability to resist environmental actions, ensuring the normal service life of building structures. Therefore, in the process of researching and designing concrete materials, improving mechanical properties should be accompanied by attention to durability. Among them, sulfate attack is one of the critical environmental factors causing durability degradation and a shortened service life of concrete [17]. The deterioration of concrete due to external sulfate erosion occurs as SO_4_^2−^ ions penetrate into the concrete through connected pores under a certain osmotic pressure along with water. The ions combine with alkali metals in the matrix, leading to sulfate crystallization expansion. Meanwhile, the ions react with cement hydration products in the concrete matrix to generate a large amount of expansive products such as ettringite and gypsum [18]. During sulfate erosion, calcium hydroxide (CH) in the matrix is consumed, and calcium silicate hydrate (C-S-H) undergoes decalcification [19,20], causing damage to the concrete matrix, leading to the expansion, cracking, and even spalling of concrete; a decrease in strength; and a severe impact on the service life of concrete structures.

More importantly, according to the China Ecological Environment Status Bulletin 2021 published by the Ministry of Ecology and Environment (MEE) [21] in 2022, there are still “relatively severe acid rain areas” in China, including parts of the Pearl River Delta in Guangdong. The acid rain remains predominantly sulfuric-type, with sulfate ions as the main anions in rainfall, accounting for 18.7% of the equivalent concentration ratio. Meanwhile, the Pearl River Delta in southern China has a subtropical marine monsoon climate with simultaneous rainfall and heat, featuring abundant summer rainfall. Studies have found that concrete structures in the Pearl River Delta experience large daily temperature differences in summer, making concrete buildings prone to dry–wet cycles during the rainy season. Combined with the acid rain environment in the Pearl River Delta, concrete is susceptible to sulfate erosion degradation under dry–wet cycles, which poses a test to concrete durability. Additionally, the evolution law of the mechanical properties and pore permeability changes of concrete at long ages need to be considered, as these are related to the overall durability of concrete during its service life.

Herein, we conducted an experimental study aimed at investigating the durability of concrete with close-packing aggregate, primarily exploring its long-term mechanical properties, water absorption, and sulfate attack resistance under dry–wet cycles. Furthermore, we analyze the effects of the close packing of aggregates, silica fume content, and volume fraction of micro-steel fibers on the concrete’s long-term mechanical properties, water absorption, and sulfate attack resistance under dry–wet cycles, and the mechanisms of these effects. This study aims to provide a theoretical basis and engineering reference for scientific research and practical applications for concrete with close-packing aggregate.

## 2. Materials and Methods

### 2.1. Characteristics of Experimental Materials

High-grade cement served as the main cementitious material. In this study, P.Ⅱ52.5 cement produced by Guangdong Yangchun Conch Cement Co., Ltd. (Yangjiang, China). was selected. Table 1 presents the chemical composition of the cement. Silica fume was procured from Gansu Sanyuan Silicon Materials Co., Ltd. (Lanzhou, China). The gradation and packing characteristics of coarse and fine aggregates are decisive factors affecting the overall elastic modulus of concrete. The coarse aggregate used was granite crushed stone from Jinxing Quarry in Jiangmen, Guangdong, with particle sizes of 5–10 mm and 10–25 mm. The fine aggregate was natural river sand from Beijiang Sand Field in Qingyuan, Guangdong. Short straight copper-plated micro-steel fibers provided by Shanghai Zhenqiang Fiber Co., Ltd. (Shanghai, China). were used to reinforce and toughen the high-elastic-modulus concrete. The concrete superplasticizer adopted was a polycarboxylic acid-based high-performance retarding superplasticizer from Sika (Jiangsu) Building Materials Co., Ltd. (Zhenjiang, China). Potable tap water from Guangzhou was used as mixing water.

### 2.2. Mixing Procedure and Specimen Preparation

This research developed concrete mixtures that included steel fibers as agents that increase toughness and silica fume as an additional cementitious element, building on the concepts of the dense packing of coarse particles. The coarse aggregates used in the tests were notable for having large particle sizes (up to 25 mm, 1 in.), which made it necessary to limit the amount of steel fiber in order to prevent clustering from poor dispersion and subsequent void formation—defects that would seriously impair the concrete’s mechanical and durability qualities [22]. In order to increase toughness, modest dosages of micro-fine steel fibers were chosen.

In this study, 13 concrete mix designs were created: A0 was the control (C50-grade plain concrete with a cube compressive strength ≥ 50 MPa), and B1–B12 used the dense packing design system for coarse aggregates, modified with micro-fine steel fibers (0%, 0.2%, 0.4%, and 0.6% by volume) and silica fume (0%, 2%, and 4% by mass). This study examines the effects of steel fiber and silica fume dosages on different concrete qualities; Table 2 provides the specific mix proportions.

Procedure for Mixing: (1) Sand, silica fume, cement, and coarse aggregates are dry mixed for two minutes to ensure a homogeneous and even mixture. (2) They are mixed for a further two minutes after adding the pre-mixed superplasticizer solution with water. (3) Steel fibers are added, and the mixture is stirred quickly for four minutes at high speed to guarantee even dispersion.

In order to simulate in situ building circumstances, this study used typical wet covering curing techniques that are frequently employed in engineering. The following was the particular cure procedure: Fresh concrete’s exposed surfaces were securely covered with geotextile fabric, which was sprayed with water on a regular basis to keep the material moist. After applying this wet covering curing for seven days, the specimens were allowed to naturally cure at room temperature until the testing age, at which point the pertinent concrete parameters were examined.

### 2.3. Experimental Test Details

According to the Testing Methods of Cement and Concrete for Highway Engineering [23], the concrete compressive strength test was carried out.

The concrete water absorption test was conducted using 100 mm × 100 mm × 100 mm standard cube specimens in accordance with the Standard for Test Methods of Concrete Physical and Mechanical Properties [24]. The particular test methods were as follows:

(1) At the testing age, specimens were submerged in water that was kept at (20 ± 2) °C. The lower portions of the specimens were raised so that the water surface was at least 25 mm over the tops of the specimens. Specimens were removed from the water after 24 h, their saturated surface-dry masses were weighed, and surface moisture was removed using a wrung-out damp cloth. After that, the specimens were submerged once more for 24 h, repeating the previous procedures. The immersion was stopped when the mass change between two consecutive 24 h immersion intervals was less than 0.2% of the bigger value, after it had been continuing for at least 48 h. It was noted that the final saturated surface-dry mass was m_s_.
(2) The samples were put in a drying oven with forced air that was kept at (105 ± 5) °C. Following a 24 h drying period, the samples were removed and allowed to cool to ambient temperature in a desiccator, and their oven-dry masses were measured. Following that, the drying procedure was repeated for a further twenty-four hours. After at least 48 h, the drying process was stopped when the mass change between two 24 h drying intervals was less than 0.2% of the smaller value. It was noted that the final oven-dry mass was m_d_.

Equation (1) below was used to determine the concrete’s water absorption rate following the measurement of the specimens’ saturated surface-dry mass (m_s_) and oven-dry mass (m_d_) using the procedures described above:

(1) $$W_{a}=\frac{m_{s}-m_{d}}{m_{d}}\times 100\%$$

where W_a_ is the water absorption rate of concrete (%); m_s_ is the saturated surface-dry mass of the saturated specimen (g); m_d_ is the oven-dry mass of the specimen (g).

This study used 100 mm × 100 mm × 100 mm cube specimens to perform the sulfate attack test in compliance with the Standard for Test Methods of Long-term Performance and Durability of Ordinary Concrete [25]. A 5% Na_2_SO_4_ solution was used to create the sulfate environment, and alternating specimen immersion and drying was used to replicate the dry–wet cycles, simulating the concrete service conditions under coupled dry–wet cycles and sulfate erosion. Here are the particular test methods:

(1) After being cured for 26 days, the test specimens were taken out, the surface moisture was wiped off, and they were then kept in an oven set at (80 ± 5) °C for 48 h. They were then allowed to cool to ambient temperature in a dry setting after drying.
(2) The cooled specimens were submerged in a 5% Na_2_SO_4_ solution that had been prepared, with the solution level at least 20 mm above the specimen surfaces. After 15 h of soaking, the specimens were taken out and allowed to air dry for one additional hour. After that, they were put in an oven that was set at (80 ± 5) °C and left there for six hours to dry. Following drying, the samples were taken out and allowed to cool for two hours before being submerged once again in the 5% Na_2_SO_4_ solution to finish a 24 h dry–wet cycle.

It should be mentioned that measurements were performed every 15 cycles to keep the pH of the Na_2_SO_4_ solution between 6 and 8, and that the pH value was routinely checked and corrected.

The sulfate resistance of concrete tested with 15, 30, 60, 90, 120, and 150 dry–wet cycles was examined in this study. The concrete mass (m) and compressive strength (f_c_) are test parameters. To quantitatively assess the sulfate resistance of concrete under dry–wet cycling, the relative mass change rate (K_m_) and compressive strength corrosion factor (K_c_) were computed by comparing these parameters with those of normally cured concrete at the same age. Equations (2) and (3), respectively, provide the formulae for K_m_ and K_c_.

(2) $$K_{m}=\frac{m_{n}-m_{0}}{m_{0}}\times 100\%$$

where K_m_ is the relative mass change rate (%), which is the percentage change in mass; m_n_ is the mass (kg) of the concrete specimens exposed to sulfate attack following N dry–wet cycles; m_0_ is the mass (kg) of the control concrete specimens at the same age under normal curing conditions.

(3) $$K_{c}=\frac{f_{\mathrm{cn}}}{f_{c0}}\times 100\%$$

where K_c_ is the compressive strength corrosion factor (%), which is defined as the ratio of compressive strengths; f_cn_ is the compressive strength (MPa) of the concrete specimens exposed to sulfate attack following N dry–wet cycles; f_c0_ is the compressive strength (MPa) of the control concrete specimens at the same age as the test specimens.

## 3. Results and Discussion

### 3.1. Long-Term Mechanical Properties

#### 3.1.1. Long-Term Compressive Strength

**Figure 1:**
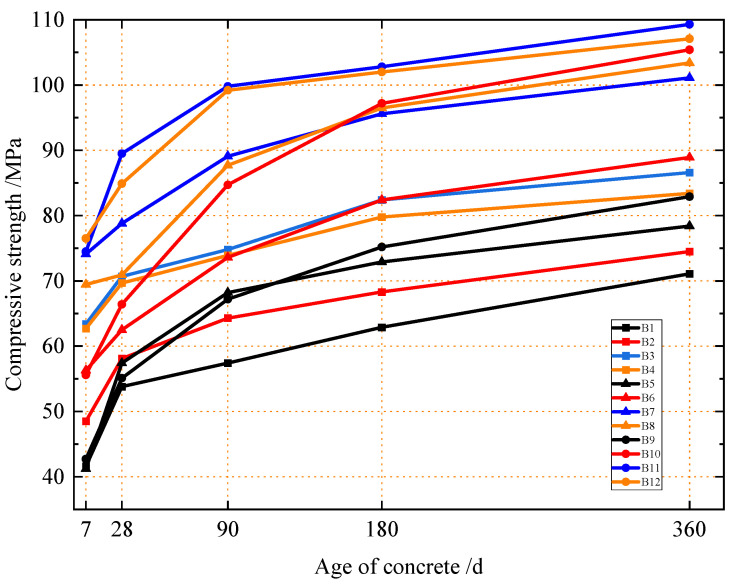
Compressive strength of concrete at different ages under different ratios.

Concrete structures typically undergo long-term service, and their safety and reliability during service require the material to possess stable long-term mechanical strength [26]. Figure 1 presents the compressive strength of concrete with varying silica fume and micro-fine steel fiber dosages at 7, 28, 90, 180, and 360 days. As observed in Figure 1, the compressive strength of all the concrete groups increased rapidly from 7 to 28 days, with an average increase of 17.5%. From 28 to 90 days, the overall growth slowed, with an average increase of 14.9%. Thereafter, the compressive strength growth gradually stabilized from 90 to 180 days and 180 to 360 days, with average increases of 8.6% and 7.6%, respectively.

Further analysis shows that, at 7 and 28 days, groups B7, B11, and B12 exhibited higher compressive strengths: the 7-day strengths were 74.1 MPa, 74.5 MPa, and 76.5 MPa, respectively, while the 28-day strengths were 78.8 MPa, 89.5 MPa, and 84.9 MPa. In contrast, groups B1, B5, and B9 had lower strengths: the 7-day strengths were 41.6 MPa, 41.2 MPa, and 42.7 MPa, respectively, with 28-day strengths of 58.1 MPa, 57.4 MPa, and 55.1 MPa. Notably, the higher-strength groups (B7, B11, B12) had higher volume dosages of micro-fine steel fibers, whereas groups B1, B5, and B9 contained no steel fibers. This indicates that steel fibers exhibit a more significant strengthening effect on the 7-day and 28-day compressive strength of concrete than silica fume, with the incorporation of micro-fine steel fibers notably improving early-age compressive performance.

Meanwhile, at later ages (90 d, 180 d, and 360 d), groups with silica fume but little or no steel fibers, such as B5, B6, B9, and B10, exhibited higher compressive strength growth rates than other groups. In particular, groups B9 and B10 with 4% silica fume exceeded the average growth rates of all the groups at each later age. For group B9, the compressive strengths at 28 d, 90 d, 180 d, and 360 d were 55.1 MPa, 67.2 MPa, 75.2 MPa, and 82.9 MPa, respectively, with growth rates of 22.0%, 11.9%, and 10.2% at each stage. For group B10, the compressive strengths at 28 d, 90 d, 180 d, and 360 d were 66.4 MPa, 84.7 MPa, 97.2 MPa, and 105.4 MPa, respectively, with growth rates of 27.6%, 14.8%, and 8.4% at each stage. This indicates that silica fume improves the later-stage compressive strength of concrete, and the enhancement effect increases with dosage [27]. It also demonstrates that silica fume continues to contribute to the development of compressive strength over the long term: its particles fill the pores of the densely packed aggregate matrix, participate in cement hydration, promote the continuous formation of C-S-H gel, fill matrix defects, increase the concrete density, and thus effectively improve the later-stage compressive strength of concrete [28].

In addition, if the 360-day compressive strength is used as the indicator for the long-term compressive strength of concrete, it can be seen from Figure 1 that, when the silica fume dosage is 4% and the micro-fine steel fiber dosage is 0.4 vol.% (Group B11), the 360-day long-term compressive strength of concrete is the highest at 109.3 MPa. Groups B12 and B10 followed, with 360-day compressive strengths of 107.1 MPa and 105.4 MPa, respectively.

#### 3.1.2. Long-Term Flexural Strength

**Figure 2:**
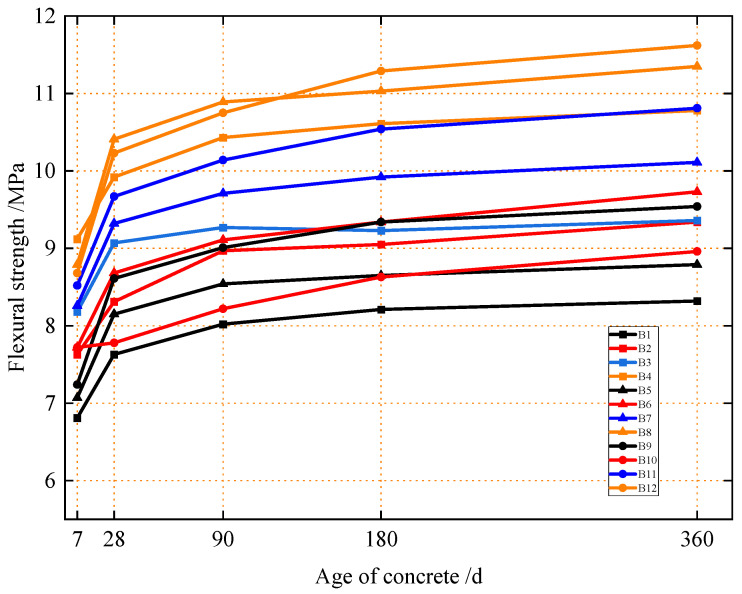
Flexural strength of concrete at different ages under different ratios.

Figure 2 presents the flexural strength of concrete with different mix proportions at 7 d, 28 d, 90 d, 180 d, and 360 d. First, an analysis of Figure 2 indicates that the long-term development trend of concrete’s flexural strength is similar to that of its compressive strength, which can be divided into three stages: (1) Stage I: 7 to 28 days. During this stage, the flexural strength of all the concrete groups increases rapidly, with an average growth rate of 12.6%. (2) Stage II: 28 to 90 days. In this stage, the growth rate of the flexural strength of each concrete group decreases, the growth slows down, and the average growth rate of the strength is 4.9%. (3) Stage III: 90 to 180 days and 180 to 360 days. At this time, the flexural strength of the concrete grows slowly, and the growth rate tends to be flat. With the increase in age, the average growth rate of the concrete’s flexural strength is 2.5% in both periods.

Moreover, observation of Figure 2 shows that the long-term flexural strength of concrete is significantly positively correlated with the dosage of micro-fine steel fiber but shows no obvious correlation with the dosage of silica fume. Groups B4, B8, and B12 with 0.6 vol.% micro-fine steel fiber exhibited significantly higher flexural strength at all ages than other groups, with 360-day flexural strengths of 10.78 MPa, 11.35 MPa, and 11.62 MPa, respectively. Next were Groups B7 and B11 with 0.4 vol.% micro-fine steel fiber, with 360-day flexural strengths of 10.11 MPa and 10.81 MPa, respectively. As shown in Figure 2, Group B12 (with 4% silica fume and 0.6 vol.% micro-fine steel fiber) achieved the highest 360-day long-term flexural strength of 11.62 MPa, followed by Group B8 with 11.35 MPa.

### 3.2. Water Absorption Rate

The water in the environment acts as a transmission carrier for aggressive ions and carries the aggressive ions to penetrate into the concrete continuously, which is the main reason for the deterioration of concrete. Research scholars believe that the key factor affecting the durability of concrete is the permeability of the concrete [29,30,31]. The water absorption of concrete is a common index for testing the watertightness of concrete, which can reflect the internal structure of concrete such as capillary pores, fine cracks, and connected pores [32,33,34]. The magnitude of water absorption can effectively characterize the permeability performance of concrete. Therefore, this section investigates the water absorption rate to provide a reference for the durability performance of concrete.

**Figure 3:**
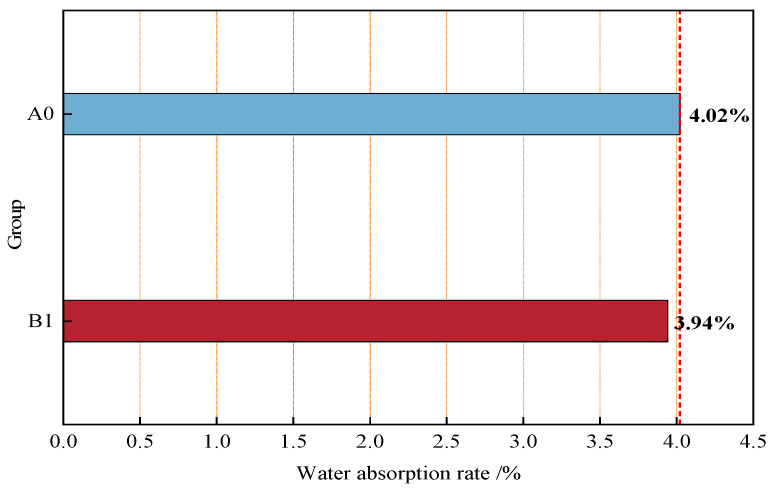
Comparison of 28-day water absorption rates of concrete after close-packed of benchmark concrete and aggregates.

Figure 3 shows the 28-day water absorption results of the control group A0 and Group B1 with close-packed aggregates. As indicated in Figure 3, the water absorption of Group B1 (with optimized close-packed aggregates) is 3.94%, slightly lower than that of the control group A0 (4.02%), representing a 2.0% reduction.

The close-packed aggregate system reduces water absorption because the densely packed aggregates form a compact concrete skeleton, which optimizes the internal structure of the material, partially reducing pathways for water penetration into the matrix and thus slightly lowering water absorption. However, water absorption is primarily influenced by microstructural defects such as capillary pores, interconnected voids, and microcracks, while the dense skeleton only modifies the concrete’s internal structure at the macroscopic level [35]. Therefore, the effect of close-packed aggregates on water absorption is not significant.

**Figure 4:**
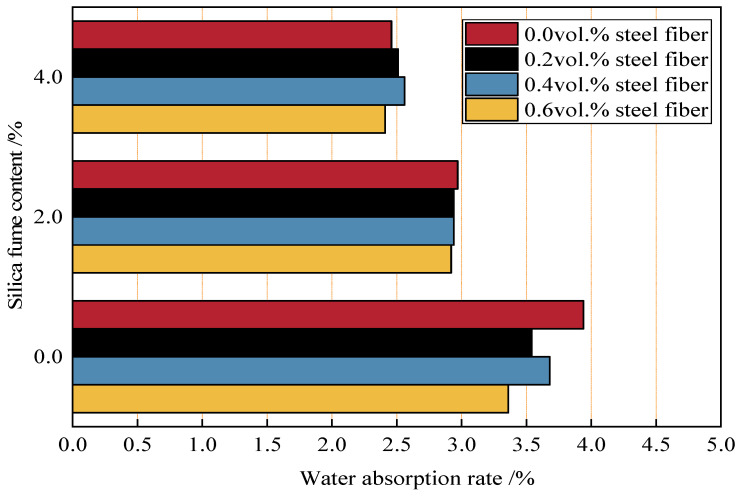
The 28-day water absorption rate of concrete at different ratios.

Figure 4 presents the 28-day water absorption of concrete with close-packed aggregates containing varying dosages of silica fume (SF) and micro-fine steel fiber (MSF). First, as observed in Figure 4, when only MSF was added, the water absorption of concrete decreased with increasing MSF volume dosage. Compared to Group B1 (0 vol.% MSF), Groups B2, B3, and B4 (with 0.2 vol.%, 0.4 vol.%, and 0.6 vol.% MSF) exhibited 10.2%, 6.6%, and 14.7% lower water absorption, respectively. This is because micro-fine steel fibers fill micron-scale pores in the close-packed aggregate matrix; meanwhile, the fibers intersperse within the matrix, connecting it to reduce microcracks, minimizing internal interconnected pathways for water absorption, improving the overall water tightness of the concrete, and thus lowering water absorption.

Figure 4 also shows that, when only SF was added, Groups B5 and B9 (with 2% and 4% SF) had 24.6% and 37.6% lower water absorption than Group B1, respectively. Water absorption decreased significantly with increasing SF dosage, with a more pronounced effect at higher dosages. This is attributed to the pozzolanic effect of SF, which promotes cement hydration to generate abundant C-S-H gel, enhancing the compactness of the concrete matrix. Additionally, due to the micro-aggregate effect of SF, its particles further fill micro-nano pores in the concrete, substantially reducing internal micro-pores and capillary pores, interrupting most water penetration pathways, and thus significantly reducing water absorption [36].

In addition, Figure 4 further reveals that, when silica fume is incorporated into the concrete, increasing the volume dosage of micro-fine steel fibers no longer exerts a noticeable impact on reducing water absorption. In contrast, increasing the silica fume dosage continues to bring about a significant decrease in water absorption, which indicates that silica fume has a more potent effect on lowering water absorption.

The reason for this lies in the fact that, in comparison with the pore-filling function of micro-fine steel fibers in addressing concrete defects, the pozzolanic effect and micro-aggregate effect of silica fume can more effectively improve the internal structure of concrete at the micro- and nano-scales. This improvement reduces internal defects and water permeation paths, thereby significantly lowering the water absorption. This also indirectly demonstrates that silica fume can effectively enhance the durability of concrete.

### 3.3. Sulfate Erosion Resistance Under Wet and Dry Cycles

Sulfate attack is one of the most important environmental factors causing a deterioration in concrete durability and shortening of building service life.

**Figure 5:**
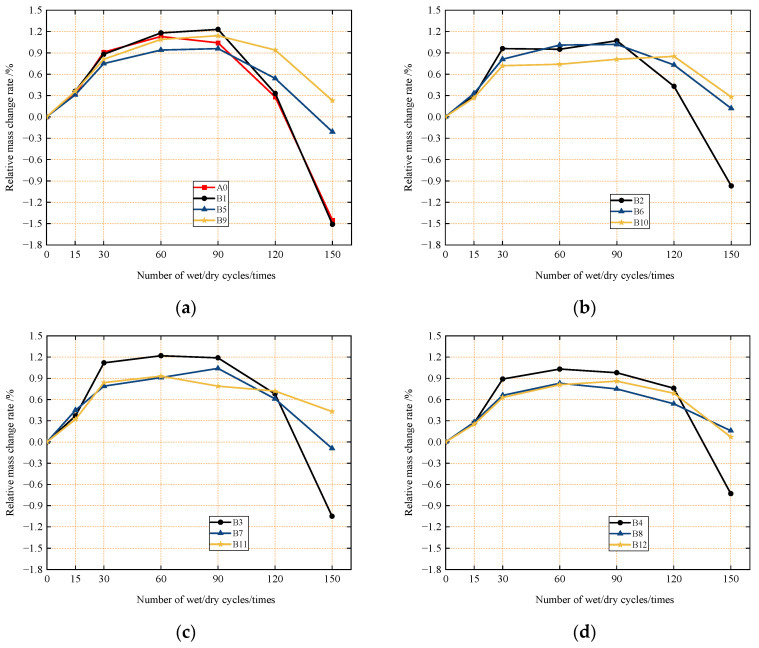
Relative mass change rate of concrete in different groups of dry and wet cycles in sulfate environment: (**a**) 0 vol.% micro-steel fibers; (**b**) 0.2 vol.% micro-steel fibers; (**c**) 0.4 vol.% micro-steel fibers; (**d**) 0.6 vol.% micro-steel fibers.

Figure 5 indicates that, in contrast to concrete under normal curing conditions, concrete subjected to sulfate attack under dry–wet cycles displays an overall mass change trend with three distinct stages as the number of cycles increases: a period of rapid mass gain, a period of stable mass change, and a period of rapid mass loss. These three stages reflect the specific process of sulfate attack on concrete.

The rapid mass gain in the initial stage is attributed to the early phase of sulfate attack: external sulfate ions penetrate the concrete through surface pores, combine with alkali metal ions to form sulfate salts, and continuously accumulate within the concrete. During the drying process, water in the concrete evaporates rapidly, causing the accumulated sulfate salts to crystallize and precipitate, which leads to an increase in concrete mass. Additionally, because the concrete is in an environment of alternating wet and dry conditions, the sulfate salts undergo repeated cycles of crystallization. The expansive crystalline salts damage the pore structure of the concrete’s surface layer, resulting in visible pores on the surface. This accelerates the rate at which sulfate ions penetrate into the interior of the concrete, thereby causing the mass of the concrete to increase at an accelerated pace [37].

After 15 or 30 dry–wet cycles in a sulfate environment, the mass change of concrete enters the second stage: the stabilization period. By this stage, substantial sulfate ions have penetrated into the interior through surface pores; over time, they react with the cement matrix to form gypsum and ettringite—both solid-phase expansive substances. Notably, ettringite growth induces significant internal expansion stress, damaging the internal capillary pore structure and causing cement matrix components to leach into the solution. Meanwhile, continuous sulfate ion infiltration promotes further formation of gypsum and ettringite. These two processes—sulfate ingress (increasing mass) and matrix leaching (reducing mass)—offset each other, resulting in relatively stable overall mass during this stage [38].

Beyond 60 or 90 dry–wet cycles, the mass of concrete in the sulfate environment begins to decrease, with the rate accelerating as cycles increase. In this rapid mass loss stage, repeated dry–wet alternation leads to the accumulation of expansive erosion products (ettringite and gypsum) in the matrix, causing expansion stress to escalate. This drives the continuous formation of new cracks and pore defects, reducing sulfate resistance. Such structural deterioration manifests as surface spalling and localized corner damage, directly causing a decline in relative mass [38,39].

In addition, Figure 5a shows that the relative mass change rates of the control group A0 and the close-packed aggregate group B1 under sulfate attack during dry–wet cycles are similar, with nearly identical trends. This indicates that close-packed aggregate design alone cannot effectively improve concrete’s resistance to sulfate attack. Comparative analysis of Figure 5a–d reveals that the incorporation of micro-fine steel fibers can reduce the magnitude of mass loss during the 90th or 120th cycle phase. This is because steel fibers in concrete mitigate crack propagation caused by expansion stresses from corrosive substances, slowing the erosion rate and preventing rapid spalling [22,40,41]. However, when the number of cycles reaches 150, the interface between concrete and micro-fine steel fibers fails, rendering the fibers ineffective and causing rapid mass loss.

Meanwhile, Figure 5a–d also show that increasing the silica fume content effectively mitigates sulfate-induced mass loss. After 150 cycles, the four groups without silica fume exhibited severe mass loss, with relative mass change rates ranging from −1.51% to −0.73% (all negative values). In contrast, the four groups with 4% silica fume had relative mass change rates of 0.19% to 0.43%, indicating improved mass loss resistance with higher silica fume content. This is because silica fume refines the concrete’s microstructures, reduces ion penetration pathways, and slows sulfate ion diffusion into the matrix in a sulfate environment. Additionally, silica fume enhances the matrix’s mechanical properties, enabling it to resist expansion stresses from sulfate crystallization or ettringite formation, thereby reducing damage and mitigating mass loss [28].

To characterize the effect of sulfate attack under dry–wet cycling on concrete’s mechanical properties, the compressive strength of specimens subjected to sulfate attack was tested and compared with that of age-matched normally cured specimens. The compressive strength corrosion resistance coefficient (K_c_) was then calculated.

**Figure 6:**
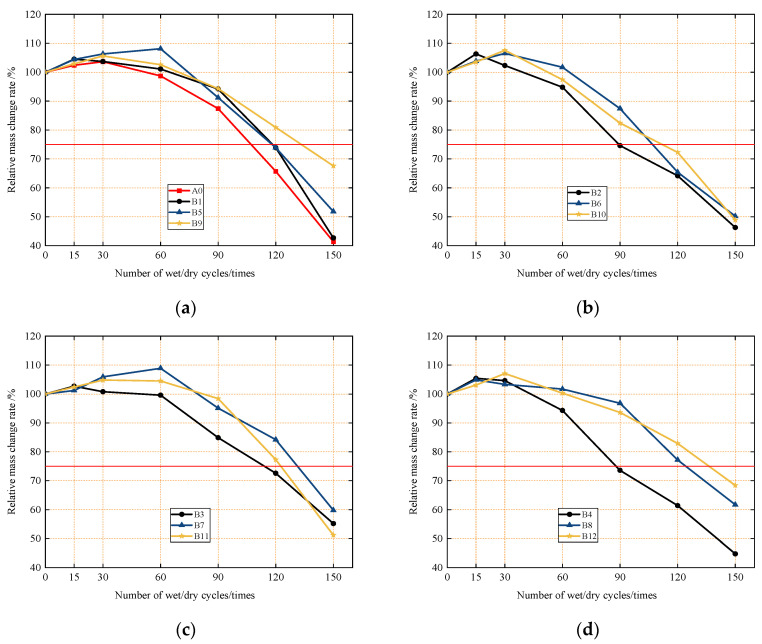
The 28-day water absorption of concrete at different ratios: (**a**) 0 vol.% micro-steel fibers; (**b**) 0.2 vol.% micro-steel fibers; (**c**) 0.4 vol.% micro-steel fibers; (**d**) 0.6 vol.% micro-steel fibers.

First, Figure 6 shows that, with increasing numbers of dry–wet cycles, the compressive strength of sulfate-attacked concrete exhibits two distinct stages of development: an initial increase, followed by rapid deterioration in the middle and later stages.

Specifically, after 15 or 30 dry–wet cycles in a sulfate environment, the compressive strength of the concrete is higher than that of normally cured concrete of the same age, with an increase ranging from 0.8% to 7.6%. This initial strength increase is attributed to sulfate ions infiltrating the concrete: during drying, substantial sulfate accumulates, and sulfate ions gradually penetrate deeper to react with cement hydration products, forming ettringite and gypsum. At this stage, the expansion stress from these expansive substances is insufficient to damage the internal structure; instead, they fill capillary pores, refine internal defects, and improve the interfacial transition zone between the cement matrix and aggregates. This makes the concrete interior denser, reduces initial defects and cracks, and thus increases compressive strength in the early stages of sulfate attack.

Subsequently, as the number of dry–wet cycles increases, the compressive strength of concrete begins to deteriorate due to sulfate attack, with deterioration becoming more pronounced as cycles continue. This rapid deterioration in compressive strength is similar to the cause of rapid relative mass loss: with increasing dry–wet cycles, sulfate continuously attacks the concrete, accumulating large amounts of expansive corrosion products until the internal pore volume reaches the critical filling threshold.

Expansive substances like ettringite generate tensile stress on the walls of concrete pores; this tensile stress from expansion rises rapidly, exceeding the concrete’s tensile strength, damaging the internal structure, and causing cracks to propagate while new cracks and pore defects form continuously—leading to decreased compressive strength. Additionally, increased permeability accelerates sulfate ion infiltration from the external environment, speeding up sulfate attack and creating a vicious cycle of corrosion. When cracks interconnect, compressive strength drops sharply.

Figure 6a shows that, in the early to middle stages of sulfate attack under dry–wet cycles, Group B1 (close-packed aggregate design) has a slightly higher compressive strength corrosion resistance coefficient than the control group A0. This is because, in these stages, the attack had not damaged the bond between the cement matrix and aggregate, so the rigid skeleton from close packing still enhanced compressive strength. In the later stages, however, erosion weakened this bond, causing B1’s compressive strength to drop rapidly.

Notably, the corrosion resistance coefficients of A0 and B1 show consistent trends, indicating that close-packed aggregates neither hinder sulfate attack nor effectively improve sulfate resistance.

Furthermore, comparative analysis of Figure 6a–d shows that, during sulfate attack under dry–wet cycles, the incorporation of micro-fine steel fibers and increased dosage have no obvious effect on the development trend of concrete compressive strength.

For concretes with the same micro-fine steel fiber dosage, those with different silica fume contents exhibit varying compressive strength corrosion resistance coefficients under the same number of dry–wet cycles—with higher silica fume content generally corresponding to higher coefficients, especially in the middle and late stages of sulfate attack. For example, Groups B4 (0% silica fume), B8 (2% silica fume), and B12 (4% silica fume), all with 0.6 vol.% micro-fine steel fibers, exhibited compressive strength corrosion resistance coefficients of 44.8%, 61.7%, and 68.4%, respectively, after 150 dry–wet cycles of sulfate attack.

This indicates that silica fume incorporation and increased dosage effectively enhance sulfate attack resistance under dry–wet cycles. This is because silica fume fully exerts its pozzolanic effect and micro-aggregate effect [42,43], improving the concrete’s microstructure, strengthening the matrix, slowing compressive strength degradation under sulfate attack, and thus enhancing sulfate resistance.

The sulfate resistance grade of concrete is defined as the maximum number of dry–wet cycles until its compressive strength corrosion resistance coefficient drops to no less than 75%. Based on this, the sulfate resistance grades of concrete with close-packing aggregate with different silica fume and micro-fine steel fiber dosages were evaluated.

Except for Groups B2 and B4, which achieved the KS60 sulfate resistance grade, all the other groups reached KS90 or higher. Among them, Groups B7, B8, B9, B11, and B12 achieved the KS120 grade. Notably, Group B12 maintained a compressive strength corrosion resistance coefficient of 68.4% even after 150 dry–wet cycles of sulfate attack—the highest among all 13 test groups—exhibiting excellent sulfate resistance.

Concrete with a KS120 sulfate resistance grade can function normally for 100 years in highly severe sulfate environments and 30–50 years in extremely severe sulfate environments. This indicates that modified, toughened concrete with close-packing aggregate generally exhibits good sulfate attack resistance.

## 4. Conclusions

(1) In the state of tight aggregate accumulation, silica fume can give full play to its pozzolanic effect and micro-aggregate effect in concrete, and increasing the silica fume content can improve the long-term compressive strength and flexural strength of concrete; the 360-day long-term compressive strength of concrete is up to 109.3 MPa, and the 360-day long-term flexural strength is up to 11.62 MPa. At the same time, when compared to other concrete materials of comparable performance, the concrete with close-packing aggregate enables an effective reduction in manufacturing costs.
(2) The 28d water absorption rate of concrete with close-packing aggregate is between 2.4% and 4.0%, the improvement in the concrete water absorption rate is not obvious with the tight packing of aggregate, and the water absorption rate of concrete can be slightly reduced by fine steel fiber; the incorporation of silica fume and the promotion of its content can effectively refine the internal structure of concrete, reduce the internal defects of the concrete and the water permeability path, and reduce the water absorption rate of the concrete.
(3) Under the conditions of dry–wet cycling, the relative mass change of concrete with close-packing aggregate is eroded by sulfate, and then increases first, slows down, and then decreases with an increase in the number of dry–wet cycles. The tight packing of aggregate cannot improve the relative quality change trend of concrete, and the incorporation of fine steel fiber and silica fume can alleviate the mass loss of concrete in the early, middle, and late stages of sulfate erosion, respectively.
(4) Under the influence of sulfate erosion under the action of dry–wet cycles, the compressive strength and corrosion resistance coefficient of concrete with close-packing aggregate first increased and then decreased rapidly with an increase in the number of dry–wet cycles. The tight packing of aggregate can maintain the compressive strength of concrete slightly in the early and middle stages of sulfate erosion, but the tight packing of aggregate and the fine steel fiber cannot hinder the strength attenuation process of concrete eroded by sulfate; silica fume improves the microscopic structure of concrete because of its volcanic ash effect and micro-aggregate effect, reduces the ionic permeation path of concrete. enhances the mechanical properties of the base body, effectively improves the compressive strength corrosion resistance coefficient of the concrete, especially the corrosion resistance coefficient of the late stage of erosion, and the highest sulfuric acid salt resistance grade of concrete with close-packing aggregate reaches KS120. The concrete can withstand normal use of 100 years in a very serious sulfate erosion environment and normal use of 30~50 years in an extremely serious sulfate erosion environment, and the concrete has good sulfate erosion resistance as a whole. When the material achieves a high-performance state, this can not only significantly extend its service life but also effectively reduce long-term operation and maintenance costs, providing dual support for the long-term service and economic efficiency improvement of engineering structures.
(5) In the concrete in which aggregate is tightly packed, silica fume and fine steel fiber are concrete-modifying materials with different functions, and the two compounds blended can synergistically modify and strengthen concrete; especially, they can synergistically enhance the mechanical properties of concrete. This is mainly due to the fact that silica fume not only improves the microstructure of concrete to improve the concrete’s performance, but also can improve the interface properties between the fine steel fiber and the concrete matrix, effectively improves the interfacial adhesion between the steel fiber and the matrix, and further improves the enhancement effect of the steel fiber on the concrete’s performance. Meanwhile, a high elastic modulus can reduce the amount of steel reinforcement required in the early construction stage, thereby lowering the initial construction costs.

Microscopic morphology testing plays a crucial role in material experiments. However, this study focuses on exploring the macroscopic durability of concrete, and microscopic morphology will be further explored in subsequent research. Meanwhile, relevant tests and mechanism analyses such as drying shrinkage testing and creep testing will also be continuously advanced in future work.

Our research will further focus on the restoration of buildings and structures in some acid rain-affected areas of the Pearl River Delta in Guangdong Province, as well as practical engineering applications. This work aims to provide a scientific basis and technical reference for improvements in the durability, disease restoration, and long-term service of concrete structures in this region.

## Figures and Tables

**Table 1:** Chemical composition of the cement (%).

SiO_2_	Al_2_O_3_	Fe_2_O_3_	CaO	MgO	SO_3_
20.3	5.26	3.38	64.2	1.27	2.88

**Table 2:** Mix proportions of concrete (%).

Number	Cement	Water	River Sand	5~10 mm Coarse Aggregates	10~25 mm Coarse Aggregates	Silica Fume	Water Reducer	Steel Fiber
A0	19.4	5.6	28.1	9.3	37.3	0	0.3	0
B1	19.4	5.6	28.1	11.7	34.9	0	0.3	0
B2	19.3	5.6	27.9	11.6	34.7	0	0.3	0.2
B3	19.2	5.6	27.8	11.5	34.5	0	0.3	0.4
B4	19.0	5.5	27.6	11.5	34.3	0	0.3	0.6
B5	19.3	5.6	28.0	11.6	34.8	2.0	0.3	0
B6	19.2	5.6	27.8	11.6	34.6	2.0	0.3	0.2
B7	19.1	5.5	27.7	11.5	34.4	2.0	0.3	0.4
B8	18.9	5.5	27.5	11.4	34.2	2.0	0.3	0.6
B9	19.3	5.6	27.9	11.6	34.6	4.0	0.3	0
B10	19.1	5.5	27.7	11.5	34.4	4.0	0.3	0.2
B11	19.0	5.5	27.5	11.4	34.2	4.0	0.3	0.4
B12	18.9	5.5	27.4	11.4	34.0	4.0	0.3	0.6

The silica fume value is relative to the binder mass. The steel fiber value is relative to the total concrete volume. The other components are relative to the total concrete mass.

## Data Availability

The original contributions presented in this study are included in the article. Further inquiries can be directed to the corresponding authors.
